# Effects of Electron Beam Irradiation on Mechanical and Thermal Shrinkage Properties of Boehmite/HDPE Nanocomposite Film

**DOI:** 10.3390/nano11030777

**Published:** 2021-03-18

**Authors:** Ju Hyuk Lee, Heon Yong Jeong, Sang Yoon Lee, Sung Oh Cho

**Affiliations:** Department of Nuclear and Quantum Engineering, Korea Advanced Institute of Science and Technology (KAIST), Daejeon 34141, Korea; aragorn477@kaist.ac.kr (J.H.L.); jeong93@kaist.ac.kr (H.Y.J.); sangyoonlee@kaist.ac.kr (S.Y.L.)

**Keywords:** polymer nanocomposite, boehmite nanoparticle, electron beam irradiation, mechanical properties, thermal properties

## Abstract

Nanocomposites comprising high-density polyethylene (HDPE) and boehmite (BA) nanoparticles were prepared by melt blending and subsequently irradiated with electrons. Electron irradiation of HDPE causes crosslinking and, in the presence of BA, generates ketone functional groups. The functional groups can then form hydrogen bonds with the hydroxyl groups on the surface of the BA. Additionally, if the BA is surface modified by vinyltrimethoxysilane (vBA), it can covalently bond with the HDPE by irradiation-induced radical grafting. The strong covalent bonds generated by electron beam irradiation allow the desirable properties of the nanofiller to be transferred to the rest of the nanocomposite. Since EB irradiation produces a great number of strong covalent bonds between vBA nanoparticles and HDPE, the modulus of elasticity, yield strength, and resistance to thermal shrinkage are enhanced by electron irradiation.

## 1. Introduction

Polyethylene is widely used due to its low cost, high chemical stability, and ease of mass production relative to other polymers. High density polyethylene (HDPE), in particular, has excellent mechanical properties due to its linear nature and high crystallinity. The intrinsic properties of HDPE can be enhanced by adding inorganic fillers, such as SiO_2_ [1,2,3], TiO_2_ [4,5,6], Al_2_O_3_ [7,8,9,10,11,12,13,14,15,16], and boron nitride [17,18], as nanoparticles to form a polymer nanocomposite. The HDPE nanocomposite can then be processed into a film and be used in various industrial fields, such as those involving food packaging [5], power cable insulation [6,14,16,19], radiation shielding [17,18], and the fabrication of lithium-ion battery separators [20,21,22].

The main issues of fabricating polymer nanocomposites exist in requiring the nano-fillers to adhere well and be thoroughly dispersed within the polymer matrix. The implementation of smaller nanoparticles enhances the properties of polymer nanocomposites more potently; however, the reduction in particle size causes agglomeration to become an even greater problem. To address these issues regarding dispersion and interfacial adhesion, the surfaces of the nanoparticles are usually silanized [8,9,10,12,14,15,16,23]. Through hydrolysis and condensation, silane coupling agents have a polar end that generates covalent bonds with hydroxyl groups found on the nanoparticles. Thus, the silanization process becomes more effective with a greater presence of hydroxyl groups.

Among the possible nanocomposite filler materials, aluminum oxide—or more specifically, boehmite (AlO(OH))—has the highest hydroxyl group content, allowing the material to react readily with silane coupling agents [24]. A limited number of works have been reported on the surface modification of boehmite for the purpose of promoting compatibility with polymers, such as polyethylene [25,26,27,28,29], polypropylene [30], and polyamide [31]. These works involve the use of silanes to modify the surface of boehmite to create a boehmite/polyethylene composite by melt extrusion that exhibits enhanced physical properties.

When attempting to utilize thermoplastics, researchers have found that physical blending is suboptimal [32]. To create a thermoplastic-based nanocomposite with optimal properties, covalent bonds must form between the silane and the thermoplastic matrix. Such covalent bonds can be generated by applying a process such as the incorporation of a radical initiator [19,32]. However, the addition of a radical initiator, such as dicumyl peroxide, produces noxious methane gas [33]. An alternative method using electron beam irradiation can accomplish the same goal without the use and production of harmful chemicals, making the method very attractive [19]. Additionally, electron beam irradiation is a simple one-step process.

Electron irradiation is often used for the polymerization, crosslinking, scission-degradation, or functionalization of materials by producing radicals without the use of any chemical agents. When polyethylene is irradiated with electrons, crosslinking occurs, and thus, the physical properties of the polymer are enhanced [34,35,36,37]. Unlike other conventional procedures involving harmful chemicals and convoluted steps, in this study, a silanized boehmite/HDPE nanocomposite film was irradiated with electrons to enhance the interfacial adhesion between the nanofiller and the HDPE matrix. By enhancing the interfacial adhesion, a significant improvement in the mechanical and the thermal properties of the nanocomposite film was achieved.

## 2. Materials and Methods

### 2.1. Materials

Grade F920A high density polyethylene (HDPE) (melt flow index: 190 °C/2.16 kg 1.0 dg/min; density: 0.956 g/cm^3^) was obtained from Hanhwa Total Petrochemical Co., Ltd. (Seosan, South Korea). Boehmite nanoparticles (BAs) with an average particle size of 20 nm were obtained from SkySpring Nanomaterials, Inc. (Houston, TX, USA). The BA particles were in the form of dry powder. Vinyltrimethoxysilane (VTMS, 98% pure CAS Number 2768-02-7) was purchased from Sigma Aldrich (St. Louis, MO, USA).

### 2.2. Preparation of Nanocomposite Films

Prior to silanization, BA was dispersed in ethanol by sonication, and VTMS (5 wt%) was dissolved in diluted ethanol (95 wt%). The PH of the solution was adjusted to 4.0 using acetic acid, after which, the solution was stirred for an hour to induce hydrolysis of VTMS. The BA was added to the VTMS solution, and the resulting solution was refluxed for 24 h under vigorous stirring. The treated BA (vBA) was filtered out, then it was washed and placed in a drying oven at 80 °C for 24 h.

Neat HDPE, BA/HDPE, and vBA/HDPE films were fabricated by melt blending using a BA-11 twin screw extruder equipped with a film dispensing unit (Bautek Co., Pochen, Korea) (Figure S1). The loading levels of the nanocomposite films were set to 8 wt%. During cast film fabrication, the screws on the extruder were rotated at 400 rpm, and the temperature profile from the hopper to the die was set to 180-185-190-190-190-200-210-220 °C. The exit cross section of the T-die was 100 mm × 0.5 mm, and the temperature of the chill rolls was set to 80 °C. The film thickness was adjusted by tuning the rotational speed of the roller in the film dispensing unit. The average range of 50 keV electrons in HDPE was calculated to be 42.1 μm; thus, the films were fabricated with a thickness of 30 μm to ensure irradiation at all depths. The draw ratio—the ratio between the initial and the final cross sections of the cast films—was 25.5. The chill roll adjacent to the T-die provided lateral constraint during drawing.

### 2.3. Electron Beam Irradiation

The samples were homogeneously irradiated with a thermionic electron gun equipped with a tantalum cathode in a vacuum chamber under a pressure of 10^−6^ torr. The energy and the current density of the electron beam were set to 50 keV and 0.5 μA cm^−2^, respectively. The electron beam was circular with a diameter of 6 cm. The electron fluences delivered to the samples ranged from 10^14^ cm^−2^ to 10^15^ cm^−2^, which correspond to the irradiation times ranging from 0.5 min to 5 min, and an absorbed dose ranging from 50 kGy to 500 kGy.

### 2.4. Characterization

To examine the morphology of the sample cross sections, samples were fractured in liquid nitrogen, and the fractured surfaces were analyzed with a scanning electron microscope (SEM, SU5000, Hitachi, Tokyo, Japan).

To assess the effects of silanization and electron irradiation on nanocomposite films, a Fourier-transform infrared spectrometer (FTIR, Nicolet iS50, Thermo Fisher Scientific Instrument, Waltham, MA, USA) was used in attenuated total reflection (ATR) mode to obtain the FTIR spectra of relevant samples.

The modulus of elasticity, yield strength, and elongation at break of 100 mm × 20 mm samples were determined based on the ASTM D882-18 standard using a universal testing machine (Instron5848, Instron, Norwood, MA, USA) with a load of 5 kN at room temperature. The gauge length and crosshead speed were set to 30 mm and 50 mm/min, respectively. The samples were stretched along the longitudinal direction, and the modulus of elasticity was calculated by determining the slope of the tangent at the initial linear portion of the force–extension curve.

Thermal properties of samples were analyzed using a differential scanning calorimeter (DSC, DSC 214 polyma, Netzsch, Selb, Germany) at a constant heating rate of 10 °C/min under a constant nitrogen flow; the samples were heated from 25 °C to 200 °C. After cooling to room temperature, the samples were heated to 200 °C during the second heating cycle. The degree of crystallinity (Xc) was calculated using the equation below:

Xc = (ΔHm/Δ°Hm) × 100,(1)

where ΔHm and Δ°Hm are the melting enthalpies of PE and fully crystalline PE (293 J/g) [38], respectively.

The thermal shrinkage tests were conducted by measuring the dimensional changes of samples after the samples were placed in an oven at 135 °C for 0.5 h. Thermal shrinkage was calculated as the percent change in area after heat exposure.

## 3. Results and Discussion

### 3.1. Morphology of HDPE Nanocomposite Films

**Figure 1:**
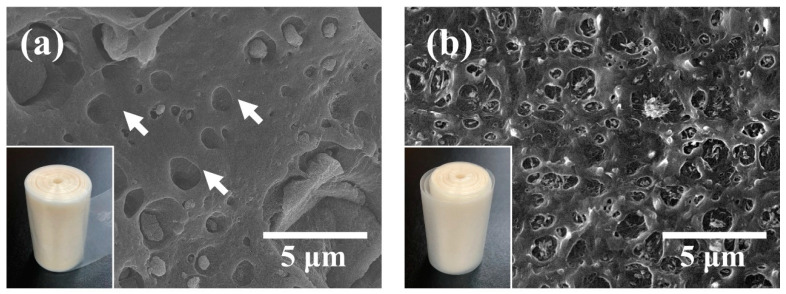
SEM images of cryo-fractured surfaces of pristine BA/HDPE (**a**) and vBA/HDPE (**b**). The insets are digital photographs of BA/HDPE and vBA/HDPE.

Figure 1 shows images of cryo-fractured cross sections of pristine BA/HDPE and vBA/HDPE nanocomposite films. With the untreated BA, the nanoparticles agglomerated to form particles that were micrometers in size (Figure 1a) due to the nanofiller having a high surface energy. The vacant cavities labeled with arrows indicate that the nanoparticles that originally occupied the cavities were displaced due to not being strongly adhered to the matrix. With the VTMS-treated BA, particle agglomeration is difficult to observe (Figure 1b). The treatment was shown to be highly effective at enhancing the dispersion property of the nanoparticles and reducing the size and frequency of vacant cavities in the HDPE matrix.

**Figure 2:**
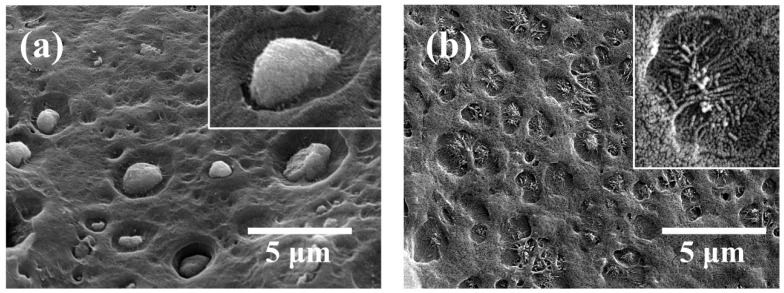
SEM images of cryo-fractured surfaces of electron-irradiated BA/HDPE (**a**) and vBA/HDPE (**b**). The insets are magnified images of the cavities formed.

Figure 2 shows images of cryo-fractured cross sections of BA/HDPE and vBA/HDPE nanocomposite films irradiated with an electron fluence of 5 cm^−2^ × 10^14^ cm^−2^. Both films showed a significant improvement in interfacial adhesion after electron irradiation; the BA nanoparticles are less prone to being dislocated from their cavities, and the needle-shaped vBA particles (Figure S2) are much more tightly bonded to the polymer matrix after irradiation. It is apparent from these observations that the electron irradiation process indeed enhances interfacial adhesion, but more so for vBA nanofiller.

### 3.2. ATR–FTIR Spectroscopy

**Figure 3:**
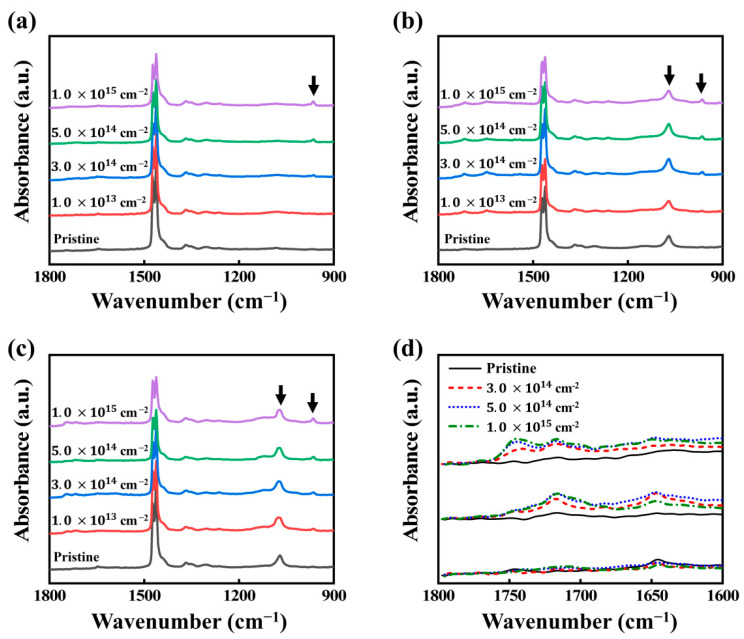
ATR–FTIR absorption spectra of (**a**) HDPE, (**b**) BA/HDPE, (**c**) vBA/HDPE before and after irradiation. (**d**) ATR–FTIR absorption spectra of irradiated HDPE, BA/HDPE, vBA/HDPE at the wavenumber range of 1800~1600 cm^−1^.

To analyze the EB-induced chemical modifications of HDPE and HDPE nanocomposites, ATR–FTIR measurements were collected. Figure 3 shows the ATR–FTIR spectra of HDPE, BA/HDPE, and vBA/HDPE before and after irradiation. Peaks corresponding to CH_2_ rocking vibration at 1463 cm^−1^ and 1473 cm^−1^ can be found in all spectra, and the electron irradiation process reduces these CH_2_ peaks and generates a new peak corresponding to trans-vinylene C=C bending at 966 cm^−1^. The peak reduction indicates the dissociation of chemical bonds and the generation of radicals, while the new peak formation can be attributed to the recombination of these radicals via crosslinking [39,40].

The addition of BA nanofiller results in the formation of the Al-O peak at 1070 cm^−1^, as shown in Figure 3b. The surface modification of these BA nanoparticles with VTMS can be detected in the ATR–FTIR spectra shown in Figure S3a and causes the particles to have enhanced dispersion property in toluene (Figure S3b). The effects of the surface modification process are difficult to detect in the ATR–FTIR spectra of vBA/HDPE due to the limited portion of the nanofiller surface affected by the surface treatment process; however, some slight changes can be observed. The Al-O peak becomes broader when the BA nanofiller is surface treated, as Si-O and Si-O-Si bonds readily form on the surface of vBA; during silanization, silanols are converted into siloxanes by condensation, while the remainder are crosslinked by EB irradiation to form even sturdier siloxane chains.

**Figure 4:**
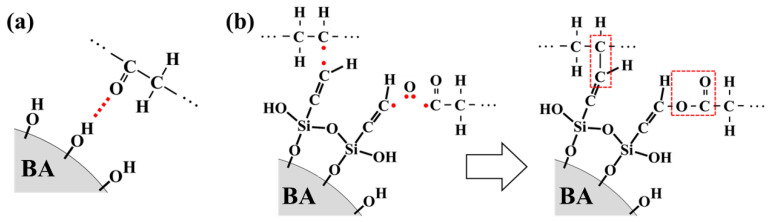
Bond formation between BA and HDPE (**a**); vBA and HDPE (**b**).

Figure 3d shows the ATR–FTIR spectra of irradiated samples. The spectra all exhibit C=O peaks at 1745, 1720, and 1645. The EB irradiation of BA can produce oxygen radicals that can react with polyethylene to form C=O bonds. In the case of BA/HDPE, the hydroxyl groups on the BA surface form hydrogen bonds with the ketone groups on HDPE. For vBA/HDPE, the vinyl functional groups found in VTMS form ester bonds with HDPE by utilizing the oxygen radicals generated during irradiation. The formation of ester bonds corresponds to the peak at 1745 cm^−1^ [41] in the ATR–FTIR spectrum of vBA/HDPE. It can be inferred from the formation of these ester bonds that C-C bonds also form from radical grafting. In other words, the vinyl group at the surface of the vBA particles can form covalent bonds with the HDPE chains via EB-induced radical grafting. The process of radical formation in HDPE by EB irradiation and the formation of bonds between the surface of the nanofiller and the HDPE matrix are depicted in Figure S4 and Figure 4, respectively.

### 3.3. Mechanical Properties

**Figure 5:**
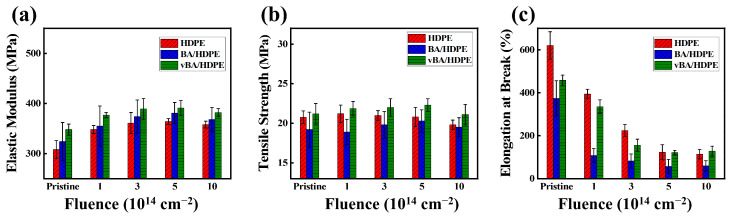
Effect of EB irradiation on modulus of elasticity (**a**), yield strength (**b**), and elongation at break (**c**) of HDPE, BA/HDPE, and vBA/HDPE.

**Figure 6:**
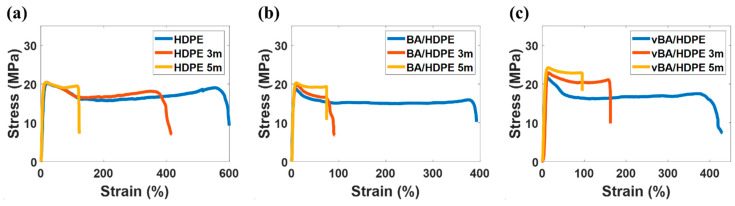
Stress–strain curves of pristine and irradiated HDPE (**a**), BA/HDPE (**b**), and vBA/HDPE (**c**).

The effects of EB irradiation on the mechanical properties of the unmodified BA and the modified BA are presented in Figure 5 and Figure 6. A significant increase in elastic modulus can be observed when BA nanofiller is added to a neat HDPE film. The BA nanoparticles restrict the chain mobility of the polymer, enhancing the strength of the polymer to resist when subjected to extension [30,42]. In particular, the modified BA provides a significantly greater boost in elastic modulus than the unmodified BA, as the former is less prone to particle agglomeration. When the nanofiller agglomerates, its ability to restrict chain mobility is notably diminished [35,37].

Electron irradiation can also serve to restrict chain mobility by crosslinking; however, irradiating past a high electron fluence of 5.0 cm^−2^ × 10^14^ cm^−2^ will induce more chain-scission than crosslinking in HDPE [43]. Thus, as can be seen in Figure 5a, the mechanical properties initially enhance as the samples are irradiated, but, past a certain point, the properties diminish. Among the samples, vBA/HDPE shows the least diminution in elastic modulus at high electron fluences due to radiation-induced bonds that are generated between vinyl radicals in VTMS and HDPE, providing another form of chain-movement restriction. vBA/HDPE also exhibits the highest yield strength among the three samples. The high interfacial adhesion of modified BA in HDPE allows stress to be transferred effectively from the HDPE matrix to the nanofiller. It can be observed that neat HDPE outperforms BA/HDPE in yield strength. Particle agglomeration—which is highly prevalent in BA/HDPE—can cause the localization of stress, creating weak spots that can diminish the overall strength of the nanocomposite.

The EB irradiation process initially decreases the elongation at break of all samples due to radiation-induced crosslinking at low doses restricting chain mobility. As chain-scission becomes more dominant at higher doses, the effects of crosslinking are negated, and the elongation property is no longer diminished. As stated earlier, the BA nanofiller provides additional chain-mobility restriction, making the two nanocomposite samples much stiffer than the neat HDPE sample. Particle aggregation, a source of stress localization, causes BA/HDPE to exhibit the lowest elongation at break among the three samples, as localized stress leads to premature tears.

### 3.4. Thermal Properties

**Figure 7:**
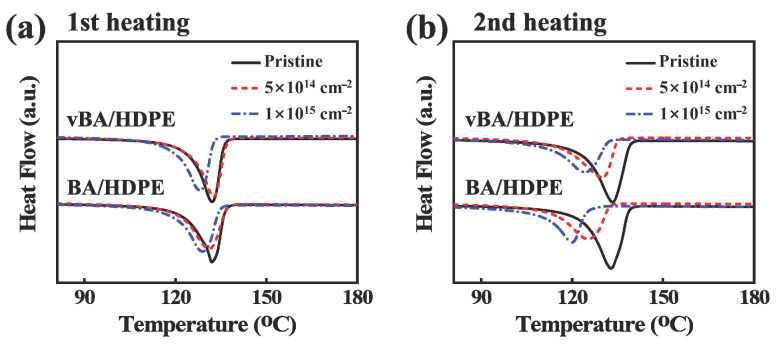
Differential scanning calorimeter (DSC) thermograms of pristine and irradiated BA/HDPE and vBA/HDPE at first heating cycle (**a**) and second heating cycle (**b**).

To assess the melting behavior of pristine and irradiated samples, DSC measurements at the first heating cycle and the second heating cycle after recrystallization were collected and shown in Figure 7 and Table 1. The melting temperature was determined by the peak position in the DSC curve during the heating cycle and was found to decrease after irradiation for all samples. The melting point is heavily dependent on the crystal state and size; thus, the decrease in melting point can be attributed to the irradiation-induced deterioration of crystallites found in the nanocomposites [44,45]. It is observable that the vBA/HDPE nanocomposite is less affected by EB irradiation in terms of melting temperature than the BA/HDPE nanocomposite. This is due to additional irradiation-induced crosslinking occurring between the vBA nanoparticles and the HDPE matrix. The DSC measurements at the second heating cycle show that the melting temperatures of irradiated samples were reduced by the initial heating process. The results indicate that the degree of crosslinking in the samples is indeed higher after EB irradiation, as crosslinking hinders recrystallization and reduces the melting temperature [35,46].

**Figure 8:**
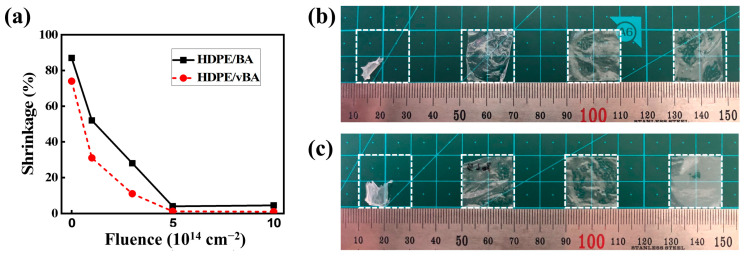
(**a**) Percentage of thermal shrinkage as a function of electron fluence. Photographs of BA/HDPE (**b**) and vBA/HDPE (**c**) exposed to 135 °C for 30 min. Degree of irradiation from left to right: pristine, 3 cm^−2^ × 10^14^ cm^−2^, 5 cm^−2^ × 10^14^ cm^−2^, and 1 cm^−2^ × 10^15^ cm^−2^.

Thermal shrinkage is closely related to the thermal stability of a polymer-based film. Reducing thermal shrinkage is crucial in applications that require high thermal stability, such as lithium-ion battery separators [20,21,22]; the separators must maintain their structural integrity at high temperatures to prevent internal short circuit. Therefore, although the operating temperature of a Li-ion battery does not exceed 60 °C, it is important to consider temperatures higher than 60 °C, as the malfunction of the battery could occur. Figure 8 shows the percentage of thermal shrinkage of the samples as a function of electron fluence. Pristine samples all underwent severe thermal shrinkage, suffering over 70% loss in surface area after being placed in an oven at 135 °C for 0.5 h. EB irradiation significantly reduced the thermal shrinkage of all samples, as irradiation-induced crosslinking drastically hinders the chain mobility of a polymer, preventing thermal shrinkage. With the vBA/HDPE sample, additional crosslinking can occur between the vBA and the polymer matrix, making the sample the least prone to thermal shrinkage.

## 4. Conclusions

The effect of electron irradiation on the mechanical and thermal properties of HDPE nanocomposite films containing BA and vBA nanoparticles were studied. HDPE, BA/HDPE, and vBA/HDPE films prepared by melt blending were irradiated with electrons under vacuum. The surface-modified nanofiller showed superior dispersion in HDPE compared to the unmodified nanofiller. Electron irradiation causes vBA nanoparticles to form strong covalent bonds with the HDPE matrix by radical grafting. A greater number of these strong covalent bonds formed results in a greater improvement of the mechanical and thermal properties of a sample. Thus, the enhancement of modulus of elasticity, yield strength, and resistance to thermal shrinkage is clearly noticeable in vBA/HDPE after electron irradiation. The applicability of the approach introduced in this work is not limited to HDPE and vBA; by carefully considering physical properties, it can be applied to other materials to achieve similar results.

## Figures and Tables

**Table 1:** The melting characteristics of pristine and irradiated BA/HDPE and vBA/HDPE.

Sample	EB Fluence (×10^14^ cm^−2^)	1st Heating			2nd Heating		
		Tm (°C)	ΔHm (J/g)	Xc (%)	Tm (°C)	ΔHm (J/g)	Xc (%)
BA/HDPE	Pristine	131.9	152.92	52.1	132.9	169.61	57.8
	5	131.1	150.81	51.4	125.3	140.96	48.1
	10	128.9	149.28	50.9	119.9	126.85	43.2
vBA/HDPE	Pristine	132.1	153.2	52.2	133.3	173.35	59.1
	5	132.6	155.5	53.1	129.8	140.64	48.0
	10	128.3	150.1	51.2	124.2	123.32	42.1

## Data Availability

No new data were created or analyzed in this study. Data sharing is not applicable to this article.

## Supplementary Materials

The following are available online at https://www.mdpi.com/2079-4991/11/3/777/s1, Figure S1: The apparatus for nanocomposite film fabrication, Figure S2: SEM images of boehmite nanoparticles, Figure S3: ATR-FTIR spectra of BA and vBA (**a**), BA and vBA dispersed in toluene for 3 days (**b**), Figure S4: Radical formation in HDPE by electron irradiation.
