# Supplementary material for: Effects of Electron Beam Irradiation on Mechanical and Thermal Shrinkage Properties of Boehmite/HDPE Nanocomposite Film
Source: Nanomaterials (Basel). 2021 Mar 18;11(3):777. doi: 10.3390/nano11030777 (PMC8003250; doi:10.3390/nano11030777)
Supplement: Supplementary file 1 [file nanomaterials-11-00777-s001.pdf]

Supplementary Materials

Effects of Electron Beam Irradiation on Mechanical and Thermal Shrinkage Properties of Boehmite/HDPE Nanocomposite Film

Ju Hyuk Lee, Heon Yong Jeong, Sang Yoon Lee and Sung Oh Cho *

Department of Nuclear and Quantum Engineering, Korea Advanced Institute of Science and Technology (KAIST), Daejeon 34141, Korea; aragorn477@kaist.ac.kr (J.H.L.); jeong93@kaist.ac.kr (H.Y.J.); sangyoonlee@kaist.ac.kr (S.Y.L.)

* Correspondence: socho@kaist.ac.kr; Tel.: +82-(0)-42-350-3823

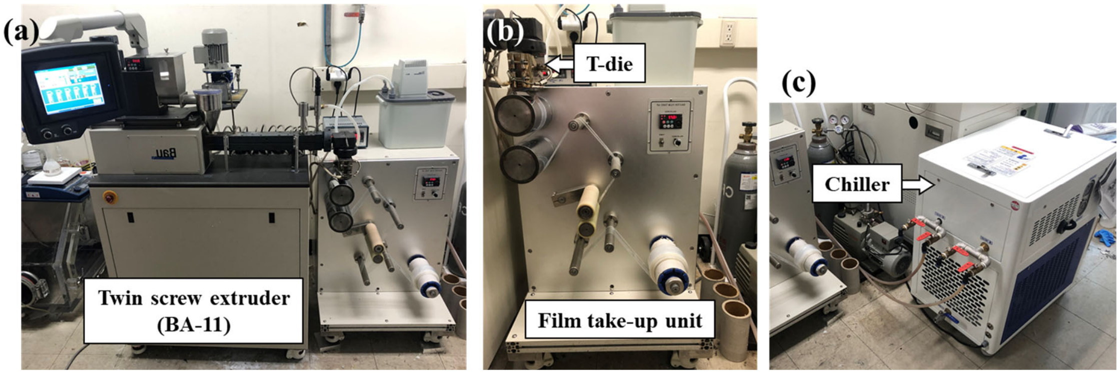

Figure S1. The apparatus for nanocomposite film fabrication.

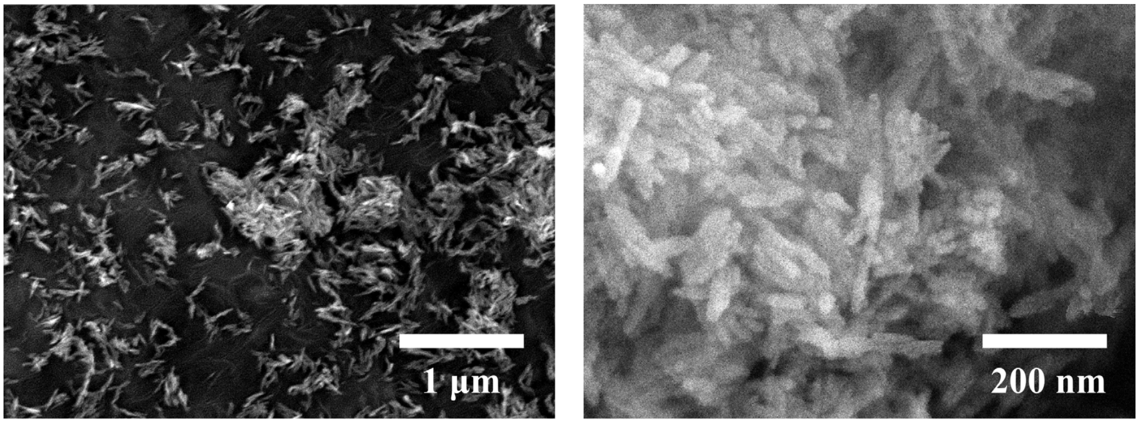

Figure S2. SEM images of boehmite nanoparticles.

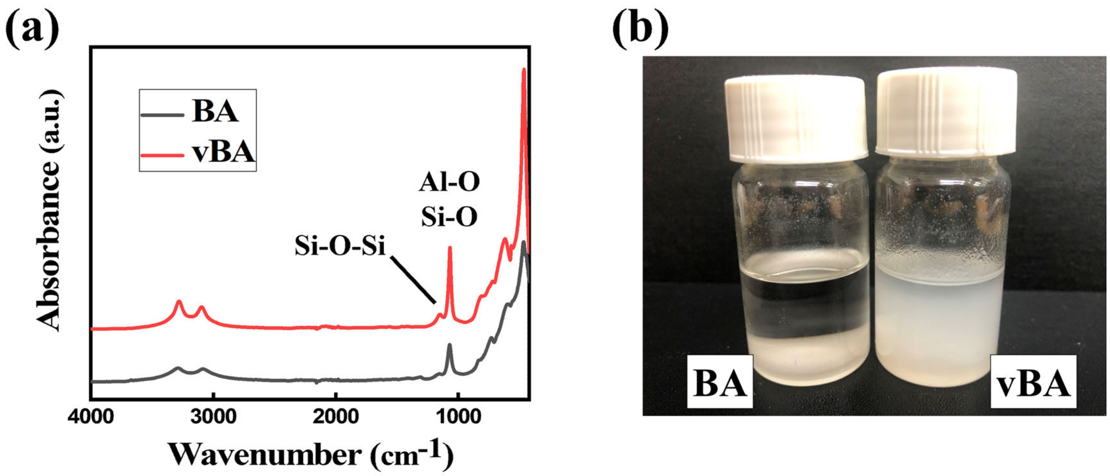

Figure S3. ATR_FTIR spectra of BA and vBA (**a**), BA and vBA dispersed in toluene for 3 days (**b**).

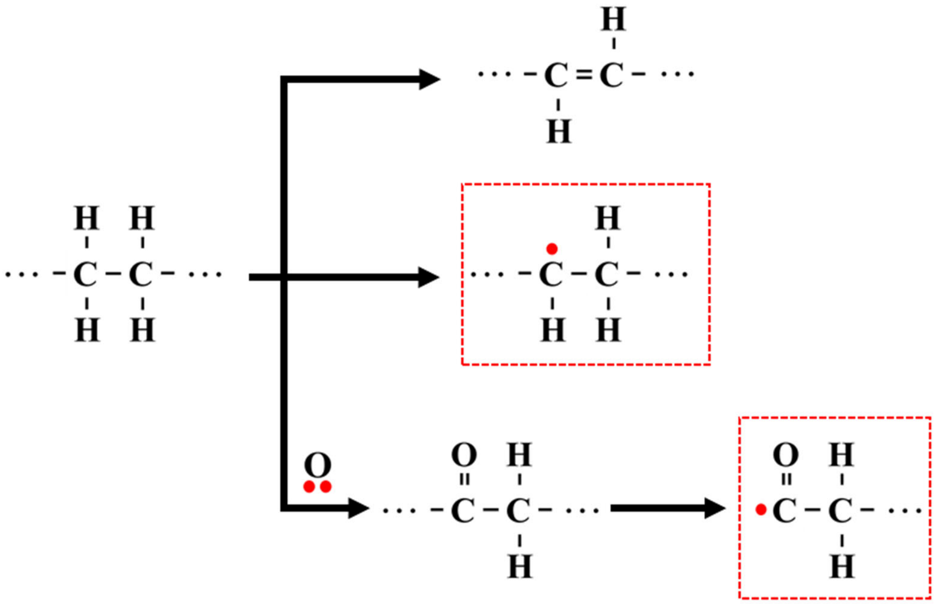

Figure S4. Radical formation in HDPE by electron irradiation.
